# Temperature Dependence and the Effects of Ultraviolet Radiation on the Ultrastructure and Photosynthetic Activity of Carpospores in Sub-Antarctic Red Alga *Iridaea cordata* (Turner) Bory 1826

**DOI:** 10.3390/plants13182547

**Published:** 2024-09-11

**Authors:** Nelso P. Navarro, Pirjo Huovinen, Jocelyn Jofre, Iván Gómez

**Affiliations:** 1 Laboratory of Ecophysiology and Biotechnology of Algae (LEBA), Faculty of Sciences, University of Magallanes, Punta Arenas 6210427, Chile; jocelynjoffre@gmail.com; 2 Network for Extreme Environments Research—NEXER, University of Magallanes, Punta Arenas 6210427, Chile; 3 FONDAP Research Center Dynamic of High Latitude Marine Ecosystems (IDEAL), Valdivia 5110566, Chile; pirjo.huovinen@uach.cl (P.H.); igomezo@uach.cl (I.G.); 4 Instituto de Ciencias Marinas y Limnológicas, Facultad de Ciencias, Universidad Austral de Chile, Valdivia 5110566, Chile

**Keywords:** photochemistry, propagules, stress tolerance, red algae, sub-Antarctic region, ultrastructure

## Abstract

The short-term effects of UV radiation and low temperature on ultrastructure, photosynthetic activity (measured as the maximal photochemical quantum yield of photosystem II: F_v_/F_m_), chlorophyll-a (Chl-*a*) contents, and UV-absorbing compounds on the carpospores of *Iridaea cordata* from a sub-Antarctic population were investigated. Exposure to both photosynthetically active radiation (PAR) and PAR + UV for 4 h caused ultrastructural modifications in all treatments. Under PAR + UV at 2 °C, a disruption of the chloroplast’s internal organization was observed. Plastoglobuli were often found in carpospores exposed to 2 °C. ‘Electron dense particles’, resembling physodes of brown algae, were detected for the first time in cells exposed to PAR and PAR + UV at 8 °C. F_v_/F_m_ decreased following 4 h exposure at 2 °C under PAR + UV (64%) and PAR (25%). At 8 °C, F_v_/F_m_ declined by 21% only under PAR + UV. The photosynthesis of carpospores previously treated with UV partially recovered after a 4 h exposure under dim light. UV-absorbing compounds were degraded in all radiation and temperature treatments without recovery after a 4 h dim light period. Chl-*a* did not change, whereas total carotenoids increased under PAR at 8 °C The study indicates that although carpospores of *I. cordata* exhibit photoprotective mechanisms, UV radiation strongly damages their ultrastructure and physiology, which were exacerbated under low temperatures.

## 1. Introduction

Once released, macroalgal propagules face a completely different physical environment than their parental reproductive structures [1,2]. Thus, propagules can be exposed to highly variable physical conditions, which could be detrimental to their development as they are more vulnerable to shifts in environmental factors compared to adult individuals [3,4,5]. However, despite the physiological machinery and regulatory processes of propagules being less developed than in adult cells, they are capable of fast acclimation to light regimes, resulting in successful recruitment [6]. Given their key importance to the establishment and fate of macroalgae populations, previous studies have examined the physiological effects of various environmental factors (especially thermal and light conditions) on propagules of macroalgae from different phylogenetic groups (e.g., [7,8,9,10]).

Considering their small size, translucent cytosol, and incipient cell wall development [11,12,13], high solar UV radiation can easily reach sensible molecules and structures inside the propagules. UV wavelengths can cause indirect and direct damage to algal cells [14]. Direct effects are mediated by UV absorption by key biomolecules such as DNA and membrane components [15], while indirect effects are mediated by the formation of reactive oxygen species (ROS). ROS induction—by exposure not only to UV-B but also to elevated PAR, UV-A, and even temperature—increases the risk of photodamage [16,17]. In photosynthetic organisms, UV-A also induces the degradation of the catalytic Mn-cluster of water-oxidizing complexes as well as D1 and D2 proteins in the reaction center of PSII [18]. Both direct and indirect effects of UV radiation, even at moderate to high levels of PAR, significantly impact photosynthetic activity [8,9,19,20,21]. These effects are not limited to biochemical levels. They can also cause visible ultrastructural changes, as demonstrated in green microalgae (e.g., *Micrasterias* spp. [22,23]) and macroalgae (e.g., red algae *Palmaria decipiens*, *Odonthalia dentata* [24,25,26], *Kappaphycus alvarezii* [27], *Iridaea cordata* [28], and green alga *Prasiola crispa* [29]). The main structures affected are lipid membranes mainly in chloroplasts and mitochondria [24,25,26,27,28,30]. This damage is mediated by the overproduction of ROS, resulting in decreased functionality, especially of photosynthetic activity [26,29]. In vascular plants [31], green microalgae such as in *Chlamydomonas reinhardtii* [32] and *Coelastrella rubescens* [33], thylakoids and/or whole chloroplasts damaged by UV exposure can suffer autophagy—a natural process of degradation in vacuoles. In this way, thylakoids and/or whole chloroplasts degradation can affect cells’ ability to absorb light energy, decreasing the risk of photooxidative damage under stressful conditions [31,34].

Photosynthetic organisms have evolved a suite of protective mechanisms against high solar radiation. The excessive energy in PAR and UV bands can be dissipated as heat [35,36], while ROS can be eliminated by enzymatic (e.g., catalase, superoxide–dismutase, and so on), and non-enzymatic (e.g., vitamins C and E, glutathione, carotenoids) antioxidants [37]. On the other hand, and at the ultrastructural level, the increase in thickness and density of cell walls in cortical cells of *Iridaea cordata* adult thalli has also been considered as a protective mechanism, with thick and dense cell walls absorbing and scattering the incident UV wavelengths [27,28,38].

UV tolerance can also be related to the presence and/or capacity to induce the formation of UV-absorbing compounds such as mycosporine-like amino acids (MAAs) [39,40,41,42] and carotenoids [36], which are effective UV shielding substances, even in small propagules [43]. UV-absorbing compounds can be induced at different UV and PAR wavelengths [44,45]. However, in the propagules of the red algae *Mazzaella laminarioides* [46] and Antarctic *Iridaea cordata* [9], exposure to a combination of PAR + UV promotes a decrease in UV-absorbing compounds. Depending on a sum of environmental and endogenous factors, temperature and UV radiation can act synergistically or antagonistically. For example, temperature can modify the susceptibility/tolerance to UV radiation in several polar and sub-polar red algal propagules [10,15]. Although previous studies have demonstrated that the photosynthetic activity of red algal spores from high latitudes is particularly sensitive to UV radiation and temperature changes [9,10,20], UV-induced changes in spores’ ultrastructure under different temperatures, particularly sublethal alterations that can impact cellular functionality, have been scarcely investigated [30].

In propagules, UV-induced ultrastructural alterations could have serious implications for metabolic functions, especially during germination, when UV radiation can reach and damage the chloroplast easily [7,21,47]. Thus, examining functional responses along with changes in ultrastructural features can provide new insights into the mechanistic organismal–environment interactions underlying the susceptibility of early stages of macroalgae to stressful conditions.

The intertidal red alga *Iridaea cordata* inhabits hard substrates in the middle–upper intertidal and tidal pools across the southern coasts of South America [48,49,50]. The phylogenetic relationship between sub-Antarctic species and Antarctic populations is under debate due to their molecular divergence [50,51,52]. Various studies carried out on the early phases of Antarctic *Iridaea cordata* have characterized some responses to environmental conditions [9,10]. Consequently, these populations likely have phylogenetically different lineages, and such responses cannot be generalized to the sub-Antarctic assemblages. Although less information is available for the sub-Antarctic population, some studies indicate a reproductive period from September to December coinciding with increasing solar radiation during the austral spring, which inhibits photosynthetic activity in haploid tetraspores [20]. Furthermore, these assemblages are exposed to temperature ranges between 2 and 10°C, which are clearly different from their Antarctic counterparts [20]. Additionally, the response to UV-B radiation from carpospores, young sporophytes, and adult thalli of *Iridaea cordata* from a sub-Antarctic population was studied [53]. In the case of carpospores, low germination and high mortality were observed under UV-B, while young and adult thalli exhibited changes in external morphology. The present study addresses the question of whether UV radiation in conjunction with low temperatures can modify the ultrastructure of carpospores of *I. cordata* from the sub-Antarctic region. Additionally, we examined the photosynthetic activity and bio-optical properties (Chl-*a* content, total carotenoids, and other UV-absorbing compounds) of carpospores exposed to UV and temperature treatment. This information could improve our knowledge of how the interaction of two key environmental factors affects the physiology of macroalgae propagules that form dominant assemblages across the Magellan region. Furthermore, it would provide valuable baseline information to project seaweed communities’ fate under global change scenarios.

## 2. Results

### 2.1. Morphological and Ultrastructural Traits of Carpospores

**Figure 1:**
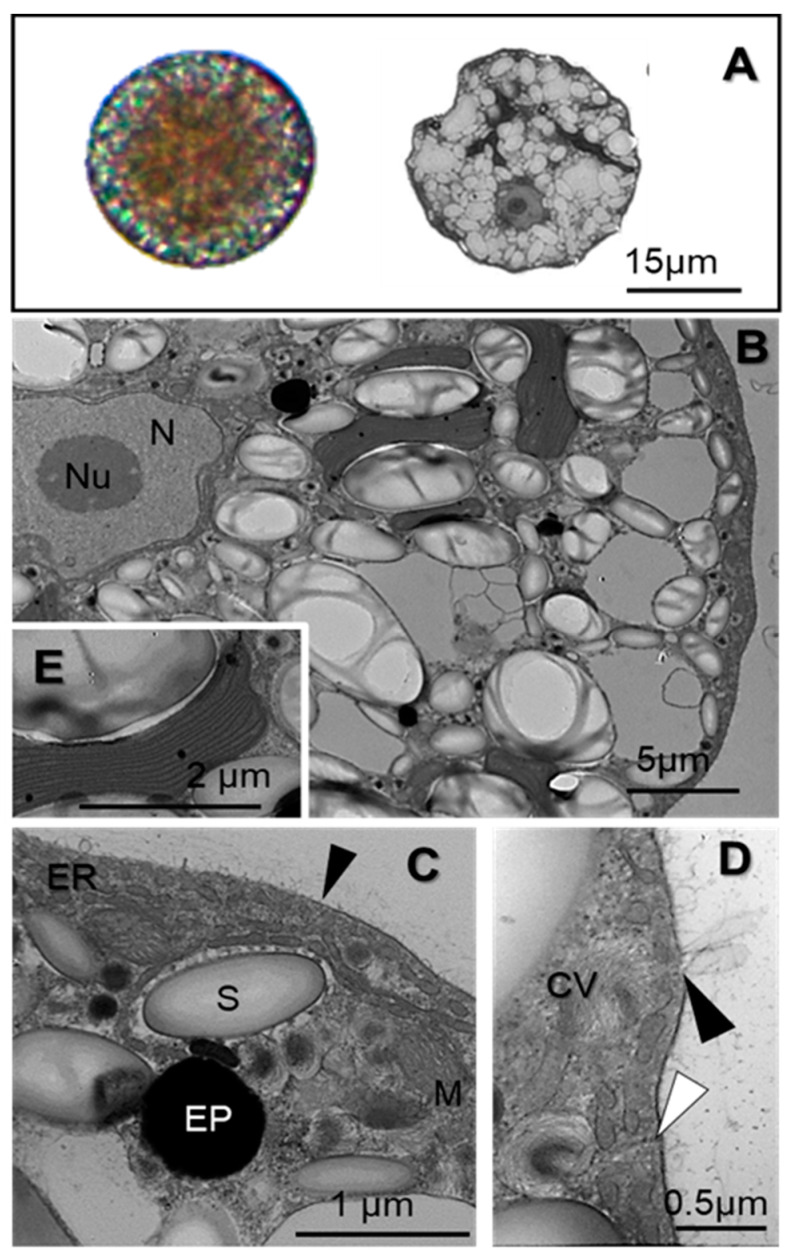
Structure of *Iridaea cordata* carpospores. (**A**) Carpospores under light microscopy and their respective ultrastructural models. (**B**–**E**) Transmission electron microscopy (TEM) of carpospores cultivated under control conditions. (**B**) Carpospores exhibit homogeneously distributed vacuolar spaces, starch grains in the cytoplasm, and a condensed nucleolus. (**C**,**D**) Thick cell walls (black arrowheads) and cored vesicles releasing their contents out of the plasmalemma; additionally, tubular invaginations (white arrowheads in (**D**) and ER are shown close to the plasmalemma. (**E**) Typical internal organization of red algae chloroplasts showing a single peripheral thylakoid. CV, cored vesicles; ER, endoplasmic reticulum; EP, electron-dense particles; M, mitochondria; N, nucleus; Nu, nucleolus.

*Iridaea cordata* carpospores averaged 30 ± 1.6 μm in diameter. The spores exhibited a reddish-green color and granular consistency under a light microscope (Figure 1A). At the ultrastructural level, freshly released carpospores are surrounded by a thick cell wall (Figure 1C,D). The spores exhibited a clear nucleus with a defined nucleolus (Figure 1B). Several floridean starch grains were scattered in the cytoplasm (Figure 1B) and electron-dense particles averaging 0.7 µm in diameter were also observed (Figure 1C). Spherical vesicles with fibrillar contents and a dense core were frequently observed close to the plasma membrane (Figure 1D). These types of vesicles resembled those reported for carposporangia in the red alga *Chondria tenuissima* called “cored vesicle” [54] as well as in cortical cells of adult thalli of *Iridaea cordata*, called cell wall-producing vesicles [28]. These vesicles were fused and released their contents from the plasmalemma (Figure 1D).

Chloroplasts had a single peripheral thylakoid, while other thylakoids were unstacked and evenly spaced (Figure 1E). Spherical to elongated mitochondria, with tubulin-like cristae (Figure 1C), were observed in close association with chloroplasts (Figure 1C). The endoplasmic reticulum (ER) surrounded the nucleus and was adjacent to the cytoplasmic membrane (Figure 1C,D). Numerous tubular invaginations varying in length were detected in close contact with the cellular membrane, which seems connected to the ER (Figure 1D).

**Figure 2:**
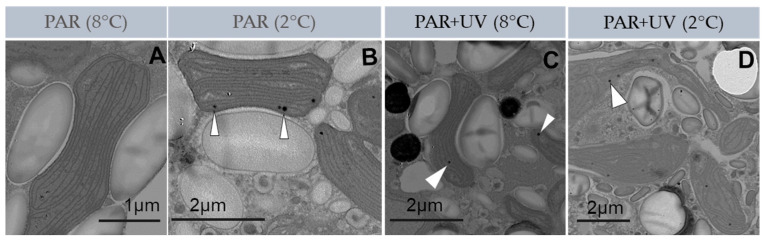
Changes in ultrastructural organization in the chloroplast of *Iridaea cordata* carpospores after exposure for 4 h to PAR (**A**,**B**) and PAR + UV (**C**,**D**) treatments at two temperatures. White arrowheads in (**B**–**D**) indicate plastoglobuli.

**Figure 3:**
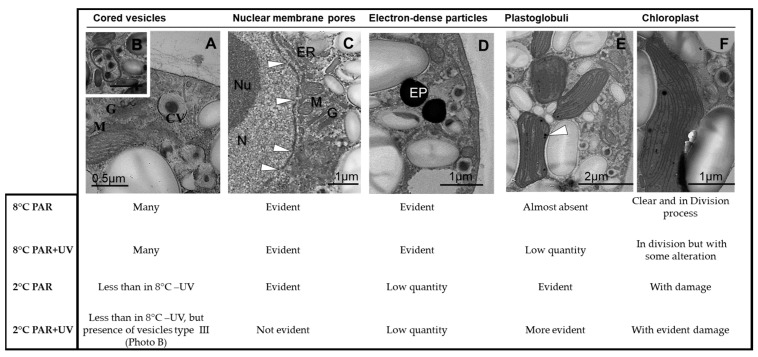
Summary of major ultrastructural changes in carpospores of *Iridaea cordata* after 4 h of exposure to PAR and PAR + UV at two temperatures. (**A**,**E**) carpospores exposed to PAR at 2 °C; (**B**) carpospores exposed to PAR + UV at 2 °C; (**C**,**D**) carpospores exposed to PAR at 8 °C; (**F**) carpospores exposed to PAR + UV at 8 °C. CV, cored vesicles; ER, endoplasmic reticulum; EP, electron-dense particles; G, Golgi complex; M, mitochondria; N, nucleus; Nu, nucleolus. White arrowheads in C indicate nuclear membrane pores, while in E plastoglobuli.

After 4 h of exposure to UV and temperature treatments, the internal chloroplast structure exhibited different degrees of alteration from almost no alteration (under PAR at 8 °C: Figure 2A) to severe damage in the thylakoid membranes (e.g., dilated and disrupted thylakoids under PAR + UV at 2 °C: Figure 2D). Under PAR + UV at 8 °C and PAR at 2 °C, damage to the chloroplasts’ structure was less evident with incipient dilatations of thylakoids (Figure 2B,C). Under PAR + UV treatment at 8 °C, abundant cored vesicles (some of them fused) were observed (Figure 3A,B). Additionally, plastoglobuli were frequently observed in the chloroplast of spores under UV radiation, mainly at 2 °C (Figure 3). In this latter treatment, starch grains were less evident. Electron-dense particles were frequently observed in the cytoplasm of carpospores at 8 °C (Figure 3D).

### 2.2. Photochemical Responses

**Figure 4:**
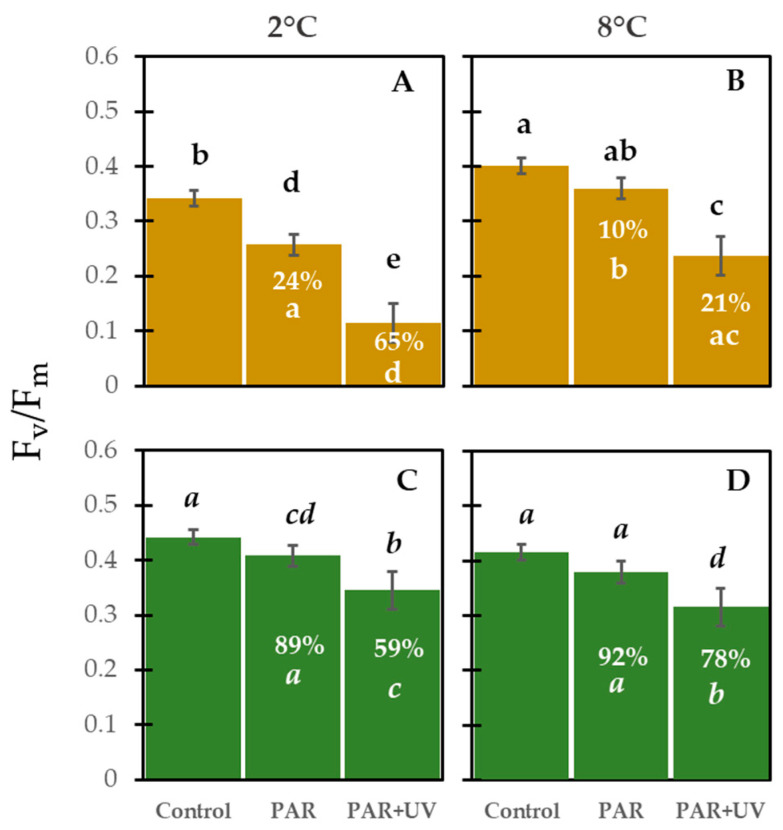
Maximum photochemical efficiency of photosystem II (F_v_/F_m_) of *Iridaea cordata* carpospores after exposure (**A**,**B**) for 4 h to PAR and PAR + UV at two temperatures and recovery (**C**,**D**) under low white light (4 μmol photon m^−2^ s^−1^). Control was continuously maintained at 4 μmol photon m^−2^s^−1^ at 8 °C (mean ± SD, *n* = 6). The percentage decrease in F_v_/F_m_ (**A**,**B**) and recovery (**C**,**D**) with respect to the control is presented within the bars. Different letters indicate significant differences (*p* < 0.05, HSD post hoc test).

F_v_/F_m_ values varied after 4 h of radiation exposure and temperature treatments (Figure 4). The two-way ANOVA showed that reduction and recovery of F_v_/F_m_ were influenced by the significant interaction between temperature and radiation treatment (Table 1). Carpospores exposed to PAR + UV at 2 °C exhibited the lowest F_v_/F_m_ values (65% F_v_/F_m_ reduction relative to the control), whereas in carpospores exposed to PAR at 8 °C, the F_v_/F_m_ declined by 10%. Considering only the temperature effect, F_v_/F_m_ decreased by 31% in carpospores exposed at 2 °C compared to those exposed at 8 °C.

After 4 h under dim light, photosynthetic recovery was partial. The F_v_/F_m_ of carpospores previously exposed to PAR + UV recovered from 59 to 78% (at 2 and 8 °C, respectively), while samples previously exposed to only PAR recovered from 89 to 92%.

### 2.3. Chlorophyll-a Content and Analysis of Absorbance Spectra

**Figure 5:**
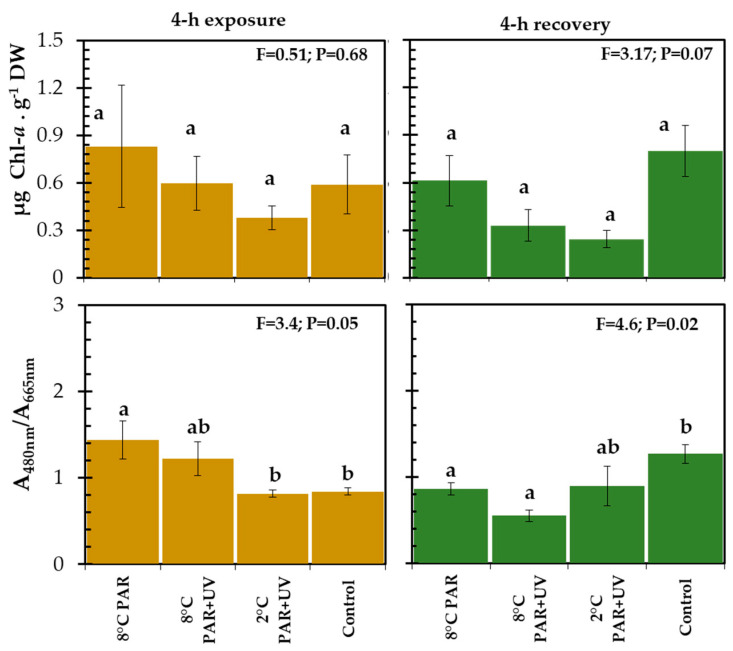
Chlorophyll *a* content in µg Chl-*a* g^−1^ DW, the ratio of carotenoids (A_480nm_) to Chl-*a* (A_665nm_) in *Iridaea cordata* carpospores exposed for 4 h to PAR and PAR + UV treatments at 2 and 8 °C, and subsequent 4 h recovery in dim light. Control was continuously maintained at 4 μmol photon m^−2^ s^−1^ and at 8 °C. Values are means ± S.E. (*n* = 4). F-values and ANOVA significance are indicated. Different letters indicate significant differences (*p* < 0.05, HSD post hoc test).

**Figure 6:**
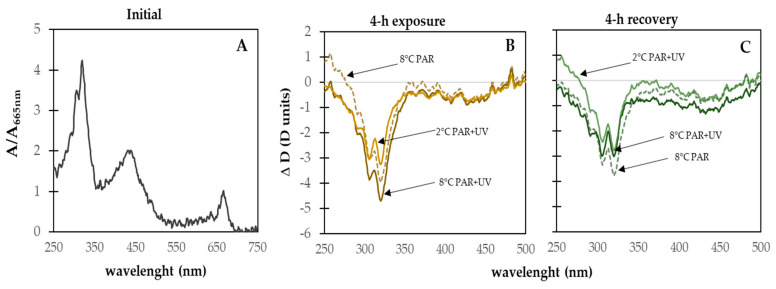
Spectra of methanol extract of the *Iridaea cordata* carpospores. (**A**) Spectra of initial samples. (**B**,**C**) Spectra of methanol extracts (against control) of samples following 4 h exposure to UV radiation under two temperature treatments and subsequent 4 h recovery in dim light. Control was kept constant at 4 μmol photon m^−2^ s^−1^ and 8 °C. Each spectrum represents the average of four measurements.

Initial Chl-*a* content reached 0.32 ± 0.26 µg Chl-*a* g^−1^ dry weight (DW). No significant decrease in Chl-*a* content was observed during the exposure (ANOVA; df = 3, F = 0.503, *p* = 0.687) and recovery (ANOVA: df = 3; F = 3.16; *p* = 0.072) period (Figure 5). To assess the presence and induction of UV-absorbing compounds and changes in pigment composition, absorbance spectra were obtained from the methanolic extract of carpospores before, during (exposure), and after (recovery) the experiments. Methanolic extracts of initial samples showed high absorbance in the UV-B and UV-A regions (peaks at 290, 305, and 320 nm), suggesting the presence of UV-absorbing compounds. Methanolic extracts of carpospores for all UV and temperature treatments were characterized by decreased absorption in the UV region of the spectrum (Figure 6B), indicating degradation of UV-absorbing compounds during the experiment (see also Figure S1: A_290nm_/A_665nm_, A_305nm_/A_665nm_, and A_320nm_/A_665nm_ ratios). After 4 h under dim light, the extracts of all treatments showed that UV-absorbing compounds were not recovered (Figure 6C). The absorbance at 480 nm (indicative of carotenoids) increased only at 8 °C in both PAR and PAR + UV treatments (A_480nm_/A_665nm_ ratio in Figure 6).

## 3. Discussions

### 3.1. Changes in Photosynthetic Activity and Ultrastructure

Photosynthetic activity declined in carpospores exposed to both light PAR + UV and PAR treatments. PAR exposure promotes decreased photosynthetic activity in shade-adapted algae. For example, propagules of shade-adapted polar and subpolar algae exhibit low saturation irradiance (E_k_) values (see data on E_k_ values of *Iridaea cordata* propagules in Supplementary Material). Additional effects on photosynthesis (11% inhibition at 8 °C) caused by UV radiation exposure and exacerbated under low temperatures (40% inhibition) agreed well with data reported for tetraspores of this species [20]. A decrease in photosynthetic activity has been widely reported in photoautotrophs exposed to UV and PAR. Temperature, however, can exacerbate or even ameliorate their negative effects on photosynthesis. Temperature dependence caused by UV-induced photoinhibition has also been evidenced in higher plants [55] and algae [20,56,57].

A decrease in photosynthetic activity in response to high irradiance is mediated by direct absorption from important components of the photosynthetic apparatus (PSA) or the lipoperoxidation action of ROS (generated by UV and even PAR wavelength exposure). Low temperatures can promote the overproduction of ROS [17], increasing the risk of damage to PSA and consequently promoting a decline in photosynthesis. On the other hand, it has been suggested that inhibition and photochemical damage are aggravated at low temperatures because of the high sensitivity of PSII-related reactions, chlorophyll turnover, and a lower repair efficiency [58]. On the contrary, photoprotective mechanisms, e.g., D1 protein turnover, enzyme repair mechanisms, and non-photochemical quenching, are activated more efficiently at higher temperatures [59,60]. In fact, F_v_/F_m_ values following recovery were lower in carpospores previously treated with UV radiation at 2 °C (59%) than those maintained at 8 °C (78%). In the same line, it is important to emphasize that elevated temperatures not only ameliorate the effects of UV during exposure periods but also can favor the recovery of photosynthetic activity of carpospores previously irradiated with UV.

Photosynthetic activity was lower in carpospores with severe alterations in their ultrastructure (PAR + UV at 2 °C). Under PAR at 8 °C, chloroplasts did not exhibit alteration, while PAR + UV at 2 °C caused disruption of thylakoids and complete alteration of the chloroplast’s internal organization, leading to decreased functionality [26,29]. Although not determined in this study, the ultrastructural alterations observed in *I. cordata* could be attributed to oxidative stress, likely mediated by the overproduction of ROS), during incubation periods [61,62,63,64,65,66]. Dilated and disrupted thylakoids are frequently observed in photoautotrophic cells exposed to high UV radiation levels. A rearrangement mechanism of membrane structures in the PSA may be responsible, similar to that observed in the autophagic process [33]. However, no evidence of autophagy was observed in the carpospores of *I. cordata*.

Carpospores exposed to 2 °C exhibited low photosynthetic activity, possibly because of changes in the chloroplast’s ultrastructural organization that diminish light harvesting while increasing plastoglobuli formation [67,68]. On the other hand, it has been suggested that a large number of plastoglobuli is strongly correlated with limited thylakoid development [64,68]. In fact, plastoglobuli formation (relevant for lipid synthesis) is observed when algae are subjected to stress [69,70] because the pathways to build protein-containing cell structures are suppressed [71].

The abundance of starch grains observed in spores under all radiation treatments suggests that the energy needed for spore attachment and germination is not supplied exclusively by photosynthesis. This trend could explain their accumulation during sporogenesis and their rapid mobilization at the initial stages of germination [72,73,74]. The presence of starch grains and an increase in the number of plastoglobuli inside the chloroplasts of cells exposed to PAR + UV radiation, mainly at 2 °C, are in agreement with observations in *Zygnema* sp. [69], *Kappaphycus alvarezii* [27], and *Chondracanthus teedei* [38]. On the other hand, the reduction in starch grains may be related to a change in the route of starch biosynthesis in the Calvin cycle, possibly by activating the degradation pathway [38].

Other ultrastructural traits such as chloroplasts in the division, ER throughout the cell, nuclear pores, and prominent nucleolus in carpospores under PAR at 8 °C could indicate intense metabolic activity in preparation for germination [75]. In the same way, tubular invaginations of the cell membrane, which were observed close to the ER. In addition, they suggest involvement in the dissemination of nutrients needed to power germination and in the transport of enzymes and cell wall compounds produced by the ER [72,74,75,76]. The presence of nuclear pores, which were less evident under UV at 2 °C, could indicate changes in the activity of the nucleus [30]. Steinhoff et al. [30] speculated that the reduced size of the nuclear pores observed in zoospores of *Laminaria hyperborea* when exposed to UV radiation would diminish the exchange rates of mRNA molecules and proteins between the nucleus and cytoplasm. However, whether the transcriptional process is altered by stress factors should be further examined. In all, the impact of UV radiation on nuclear division and translocation was previously reported for *Macrocystis pyrifera* zoospores by Huovinen et al. (2000) [7].

### 3.2. Photoprotective Compounds and Mechanisms

The presence of UV-absorbing compounds and carotenoids was evidenced in freshly released carpospores. The presence of UV-absorbing compounds and total carotenoids were also observed in spores of the sub-Antarctic red alga *Mazzaella laminarioides* [46]. Although UV-absorbing compounds were not identified in this study, the absorbance spectra could indicate MAAs, for example. The presence of two MAAs (shinorine and palythine) has been reported in spores of Antarctic species of the red alga *Sarcopeltis antarctica* (formerly *Gigartina skottsbergii*) and the cryptic species of *Iridaea cordata* [9,43]. However, in our study, absorbance spectra of methanolic extracts from spores exposed to PAR or PAR + UV at two temperatures minus control revealed that UV-absorbing compounds decreased and did not recover in any treatments agreeing with observations on spores of the red algae *Mazzaella laminarioides* [46] and the Antarctic *Iridaea cordata* [9]. The degradation of UV-absorbing compounds could supply nitrogen to damage repair after exposure to a high UV-B dose (e.g., via the production of antioxidant compounds: carotenoids?). The increase in A_480 nm_/A_665 nm_ ratios in carpospores exposed to PAR and PAR + UV at 8 °C indicates the accumulation of carotenoids, which play a crucial photoprotective role in absorbing short PAR wavelengths and UV-A radiation [36] by quenching triplet state chlorophyll molecules [77] and scavenging singlet oxygen and other toxic oxygen species formed within the chloroplast [78,79]. Thus, the presence and synthesis of total carotenoids rather than MAAs may help protect the PSA against high PAR and UV, avoiding the risk of photooxidative damage. Interestingly, the combination of low temperatures and UV seems to inhibit carotenoid synthesis. Low concentrations of carotenoids could increase the risk of photodamage in PSA, causing a decrease in photosynthetic activity (Figure 4). However, when UV radiation was excluded during the recovery period (dim light and 8 °C), a slight increase in the A_480 nm_/A_665 nm_ ratio occurred in samples previously exposed to UV and 2 °C.

On the other hand, it is important to emphasize the presence of electron-dense particles, which were frequently observed in all treatments, especially under PAR and PAR + UV at 8 °C. The appearance of these particles under transmission electron microscopy (TEM) resembles physodes, phenolic (phlorotannin)-containing structures described in brown algae [80,81]. Although the presence and significance of phlorotannins at different life stages of brown algae species have largely been recognized (reviewed by [82]), the presence of physodes in Rhodophyta has scarcely been reported. To our knowledge, the presence of structures resembling physodes has been documented in *Crassiphycus birdiae* (previously known as *Gracilaria birdiae*) under TEM [83], in *Gracilaria chilensis* using biochemical analysis [84], and in *Trematocarpus antarcticus* under violet-blue light excitation using epifluorescence microscopy [82]. Thus, phenolic compounds in Rhodophyta may be involved in photoprotection mechanisms and potentially be effective antioxidant agents, as reported for brown algae [84]. In our study, the electron-dense particles exhibited irregular shapes similar to those observed in different strains of the charophyte *Zygnema* [69,85]. However, in *I. cordata* these particles did not increase in UV-exposed carpospores as observed in *Zygnema* [69,85]. Temperature could influence their presence, as suggested for *Gracilaria chilensis* [84] and in brown algae such as *Lessonia spicata*, *Durvillaea antarctica*, and *Macrocystis pyrifera* [86]. In all, the type of compounds contained in these structures and their function in the spores of sub-Antarctic Rhodophyta are still unknown. However, the role they may play in photoprotection or initial adhesion is plausible, as has been documented in phenolics of brown algae [81].

Cored vesicles, also called cell wall-forming vesicles, observed in *I. cordata* carpospores are morphologically similar to those reported in several other red algae spores [13,72,75]. The increase in cored vesicles in carpospores treated with UV radiation at both 2 and 8 °C could imply intense polysaccharide production [54,55,56,57,58,59,60,61,62,63,64,65,66,67,68,69,70,71,72,73,74,75,76,77,78,79,80,81,82,83,84,85,86,87]. In adult thalli, an increase in these cored vesicles has been associated with an increase in the thickness and density of the cell wall, which acts as a protective mechanism for preventing or reducing the penetration of UV through cells, e.g., by increasing absorption and scattering [27,28,38]. They also suggest that these vesicles can store material for the cell wall, e.g., mucilage necessary for the attachment process. Additionally, these particles could be crystal inclusions of N reserves (guanine), which was observed in the marine dinoflagellate *Amphidinium carterae* [88].

## 4. Materials and Methods

### 4.1. The Environmental Context

In the intertidal zone and during low tide, spores of *Iridaea cordata* experience highly variable conditions of solar irradiance. Based on the Tropospheric Ultraviolet and Visible models (TUV 5.3; Madronich and Flocke [89]), the estimated summer solstice solar noon irradiances at the seawater surface (with ozone layer of 300 DU) for the study site (Bahía Mansa; Magellan Strait, 53° S; 70°59′ W) are close to 1950 μmol photon m^−2^ s^−1^ for PAR, and 1.6 and 55 W m^−2^ for UV-B and UV-A, respectively. For the winter solstice, the corresponding levels are around 350 μmol photon m^−2^ s^−1^ for PAR, and 9 and <0.1 W m^−2^ for UV-A and UV-B, respectively. Based on HOBO Dataloggers (Onset Computer Corporation, Bourne, MA, USA) placed at 2 m depth and in a pool tide in the sector of Bahía Mansa, the mean surface water temperature varied between 5 °C in winter and 8.7 °C in summer. However, in the winter, algae may be exposed to seawater temperatures close to 3 °C, while in the pools, temperatures can be as low as 0–2 °C.

### 4.2. Algal Collection and Processing

Fertile fronds of cystocarpic female gametophytes were collected from Bahía Mansa in November 2017. After sampling, algae were transferred to the Laboratory of Ecophysiology and Biotechnology of Algae at the University of Magallanes (LEBA-UMAG), where they were cleaned of epiphytes and carefully washed with filtered seawater. At least twenty disc pieces (9 cm^−2^) punched from ten different fertile fronds were subjected to dehydration for 4 h at a room temperature of 7 ± 2 °C. After rehydration for 1 h in 150 mL of filtered (0.4 μm) seawater, the carpospores were released. Carpospores were counted using a Neubauer chamber under a stereomicroscope (Carl Zeiss Microscopy GmbH, Oberkochen, Germany) before being transferred to cell culture plates (Fisher Scientific, Pittsburgh, PA, USA). Propagules were photographed under a light microscope (Primo Star; Zeiss Microscopy GmbH, Oberkochen, Germany) to obtain cell diameters.

### 4.3. Short-Term Exposure to UV Radiation

Aliquots (3 mL) from a suspension of carpospores (7.9 × 10^4^ cells mL^−1^) were placed inside cell culture plates (Fisher Scientific, Pittsburgh, PA, USA) and immediately exposed to PAR and PAR + UV treatments for 4 h at temperatures of 2 and 8 °C using a thermoregulated incubation system (Lauda Dr. R. Wobser GMBH & CO. KG, Lauda-Königshofen, Germany). This procedure was followed by a 4 h recovery period under dim light (<4 μmol photon m^−2^ s^−1^). A temperature of 8 °C matches the average seawater surface temperature recorded in the field between spring and summer in the Magellan Strait. Additionally, carpospores were also exposed to 2 °C, a temperature reflecting the extremely low temperatures at which this species can be exposed in tidal pools during winter and even early spring. The PAR and PAR + UV treatments were established using cut-off filters (Ultraphan 295 nm to obtain PAR + UV treatment, and Ultraphan 395 nm (Digefra; Munich, Germany) to obtain PAR alone) according to Navarro et al. (2019) [20]. The experimental levels of UV-B and UV-A radiation were set at 0.26 and 1.5 W m^−2^, respectively. Thus, the UV-A:UV-B ratio was lower than in natural conditions. PAR (45 μmol photon m^−2^ s^−1^) remained low to avoid masking UV effects [36]. The effect of PAR and PAR + UV radiation on photosynthesis was assessed as a decrease in F_v_/F_m_ (see below). Similarly, the effects of UV radiation at 2 °C and 8 °C on the concentration of UV-absorbing compounds and chlorophyll *a* (Chl-*a*) were assessed.

### 4.4. Determination of Photosynthetic Activity

Photosynthetic activity was determined as the maximal photochemical quantum yield of chlorophyll fluorescence of PSII (F_v_/F_m_). To accomplish this, a Water-PAM fluorometer (Walz GmbH, Effeltrich, Germany) connected to a PC with WINControl V3-36 software (Walz GmbH, Effeltrich, Germany) was used. Following a 10 min dark period, basal fluorescence (Fo) of carpospores was determined under red measuring light (approximately 0.3 μmol photons m^−2^ s^−1^), whereas maximum fluorescence (F_m_) was obtained by applying a saturating white light pulse (0.6 s; 2700 µmol photons m^−2^ s^−1^). Thus, F_v_/F_m_ was calculated as (F_m_ − Fo)/F_m_. F_v_/F_m_ was measured before (initial or time zero) and after 4 h exposure to UV and temperature treatment as well as after a 4 h recovery period under dim light.

The effect of radiation and temperature treatments on photochemistry was expressed as a percentage decrease between F_v_/F_m_ of samples treated with PAR + UV or PAR at 2 and 8 °C and F_v_/F_m_ of samples exposed to control conditions (samples maintained under <4 μmol photon m^−2^ s^−1^ at 8 °C). Additionally, the initial F_v_/F_m_ was obtained at time zero. Similarly, the recovery was estimated in samples exposed to dim light for 4 h after exposure to PAR + UV by comparing their F_v_/F_m_ values with those from control conditions.

### 4.5. Bio-Optical Traits

Before and after 4 h of PAR and PAR + UV exposure, Chl-*a* content and the presence and induction of UV-absorbing compounds and total carotenoids were determined. The Chl-*a*, carotenoids, and UV-absorbing compounds were extracted with methanol (100%) for 12 h at 4 °C in darkness. Afterward, the solution was centrifuged at 35,000× *g* for 10 min and analyzed using a spectrophotometer (280–750 nm, Spectroquant^®^ Pharo 300, Merck KGaA, Darmstadt, Germany). While the Chl-*a* content was measured using equations described in Ritchie (2008) [90], the UV-absorbing compounds, including carotenoids were determined as described by Zaytseva et al. [33] and Chekanov et al. [91]. Firstly, the absorbance spectra of treated samples were normalized using a control (A/A_665nm_−A_control_/A_665nm control_). Thus, the differential absorbance spectra (∆D(λ)) between 250 and 500 nm were estimated. An absorbance increase (compared to the control spectrum) was considered induction, whereas the decrease was regarded as loss or degradation of UV-absorbing or carotenoid compounds. Secondly, the ratios of UV-absorbing compounds (peaks at A_290nm_, A_305nm_, and A_320nm_) and carotenoids (peak at A_480nm_) to Chl-*a* (peak at A_665nm_) were analyzed.

The dry weight was determined by filtering a known volume of carpospore suspension in a 0.22 μm glass fiber filter (Merk, Millipore, Darmstadt, Germany). Afterward, the filter was dried to a constant mass in a stove at 50 °C. The difference in the weight of filters with dried cells and the weight of empty dried filters was used to determine the carpospore dry weight.

### 4.6. Transmission Electron Microscopy

After 4 h of exposure to light and temperature treatments, samples for analysis were obtained. Spore fixation in 2% glutaraldehyde and 1% paraformaldehyde in filtered seawater (0.2 μm), post-fixation in osmium–potassium ferricyanide mixture, dehydration, embedding, and infiltration followed the protocol described by Santelices et al. (1996) [92]. Cells were stained with 4% uranyl acetate and lead citrate according to Reynolds (1963) [93] and observed with a JEOL 100SX electron microscope (JEOL Ltd., Tokyo, Japan) operated at 60 kV.

### 4.7. Data Analysis

Two-way ANOVA was performed to compare the effect of temperature and radiation treatments on carpospores’ photosynthetic activity (F_v_/F_m_). One-way ANOVA was used to compare the variability in Chl-*a* concentration and ratios of UV-absorbing compounds and carotenoids to Chl-*a*. Before performing ANOVA, normality was checked using the Kolmogorov–Smirnov test. Homoscedasticity of variance was tested using Cochran tests and visual inspection of the residuals. Post hoc comparisons of means were assessed with Tukey’s HSD test. All statistical analyses were conducted using Statistica 7 software (StatSoft, Inc., Tulsa, OK, USA). 

## 5. Conclusions

UV exposure caused decreased photosynthetic activity and altered the ultrastructure of *Iridaea cordata* carpospores. While low temperatures aggravated UV-induced effects on carpospores, high temperatures favored the recovery of UV-radiated carpospores. The high UV sensitivity of *I. cordata* carpospores at low temperatures may be caused by inefficient photoprotection and repair mechanisms operating in these cells at 2 °C. The observation of electron-dense particles resembling phenolic-containing vesicles points to a possible role in photoprotection.

## Figures and Tables

**Table 1:** ANOVA results and significance values for the main effects and interactions of temperature and radiation treatments on the variation in photosynthetic activity (F_v_/F_m_) of carpospores of *Iridaea cordata* during exposure and recovery experiments.

	df	F-Value	*p*-Value	df	F-Value	*p*-Value
	F_v_/F_m_ (4 h exposure)			F_v_/F_m_ (4 h recovery)		
Radiation (A)	2	110	<0.001	2	103	<0.001
Temperature (B)	1	243	<0.001	1	75	<0.001
A × B	2	19	<0.001	2	7	0.001
	Variation of F_v_/F_m_ (4-h exposure)			Variation of F_v_/F_m_ (4-h recovery)		
Radiation (A)	1	20	<0.001	1	33	<0.001
Temperature (B)	1	7	<0.001	1	3	<0.001
A × B	1	2	<0.001	1	8	<0.001

## Data Availability

Data are contained within the article and Supplementary Materials.

## Supplementary Materials

The following supporting information can be downloaded at: https://www.mdpi.com/article/10.3390/plants13182547/s1, Figure S1. Ratio of UV-absorbing compounds (peak at A_290nm_, A_305nm_, and A_320nm_) and carotenoids (peak at A_480nm_) to Chl-*a* (peak at A_665nm_) of *Iridaea cordata* carpospores after 4 h exposure to UV radiation and subsequent 4 h recovery in dim light. Values are means ± S.E (*n* = 4). Different letters indicate significant differences (*p* < 0.05, HSD post hoc test). Table S1. Comparison of photosynthetic parameters (E_k_: [μmol photon m^−2^ s^−1^], ETR_max_: [μmol e^−^ m^−2^ s^−1^], αETR [μmol e^−^ m^−2^ s^−1^]. [μmol photon m^−2^ s^−1^]^−1^), the initial maximal photochemical quantum yield of PSII (F_v_/F_m_) and inhibition of photosynthesis by PAR and PAR + UV radiation, and subsequent recovery in spores of *Iridaea cordata* from Antarctic and su b-Antarctic populations. PAR and PAR + UV inhibition was calculated after 4 h of exposure at 2 and 8 °C, with 2 °C being the control for Antarctic individuals and 8 °C for sub-Antarctic ones. Table building considering Navarro et al. (2016, 2019) [10,20], and the present study.
