# Supplementary material for: Temperature Dependence and the Effects of Ultraviolet Radiation on the Ultrastructure and Photosynthetic Activity of Carpospores in Sub-Antarctic Red Alga Iridaea cordata (Turner) Bory 1826
Source: Plants (Basel). 2024 Sep 11;13(18):2547. doi: 10.3390/plants13182547 (PMC11435075; doi:10.3390/plants13182547)
Supplement: Supplementary file 1 [file plants-13-02547-s001.zip › plants-3165405-supplementary.pdf]

Supplementary Material for the Manuscript Entitled

Temperature dependence of the effects of ultraviolet radiation on the ultrastructure and photosynthetic activity of carpospores of the sub-Antarctic red alga *Iridaea cordata* (Turner) Bory 1826

Nelso P Navarro *, Pirjo Huovinen, Jocelyn Jofre and Iván Gómez

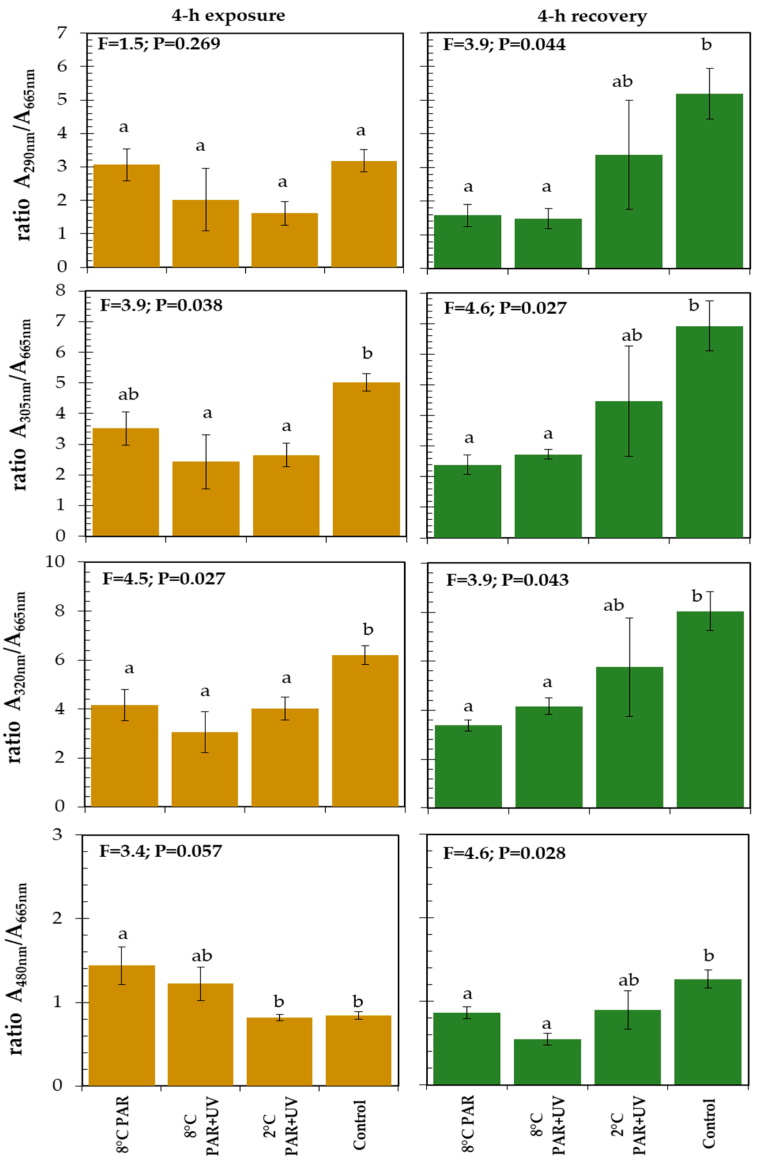

Figure S1. Ratio of UV-absorbing compounds (peak at A_{290nm}, A_{305nm}, and A_{320nm}) and carotenoids (peak at $A_{480\ nm}$) to Chl-*a* (peak at $A_{665\ nm}$) of *Iridaea cordata* carpospores after 4-h exposure to UV radiation and subsequent 4-h recovery in dim light. Values are means ± S.E (n= 4). Different letters indicate significant differences ($p < 0.05$, HSD post hoc test).

Table S1. Comparison of photosynthetic parameters (E_k: [μmol photon $m^{-2}s^{-1}$], ETR_{max}: [μmol e^- m^{-2} s^{-1}], αETR [μmol e^- m^{-2} s^{-1}]. [μmol photon m^{-2} $s^{-1}]^{-1}$), initial maximal photochemical quantum yield of PSII (F_v/F_m) and inhibition of photosynthesis by PAR and PAR + UV radiation, and subsequent recovery in spores of *Iridaea cordata* from Antarctic and sub-Antarctic populations. PAR and PAR + UV inhibition were calculated after 4 h of exposition at 2 and 8ºC, with 2ºC being the control for Antarctic individuals and 8ºC for the sub-Antarctic ones. Values from Navarro et al. (2016,2019) [10,20], and the present study.

	sub-Antarctic		Antarctic	
	tetraspores	carpospores	tetraspores	carpospores
Reproduction period	spring	**spring**	spring-summer	spring-summer
E_k	136	**167**	45	47
ETR_{max}	25	**9.1**	7	1
α_{ETR}	0.02	**0.05**	0.02	0.04
Fv/Fm	0.435	**0.41**	0.350	0.17
UV inhibition (8 and 2ºC)	36 and 67%	**21 and 65%**	no inhibition	nd
PAR inhibition (8 and 2ºC)	0 and 30%	**10 and 24%**	no inhibition	nd
Recovery from PAR	> 90%	**>90%**	100%	nd
Recovery from UV	< 75%	**<78%**	100%	nd
Temperature effect	14% decrease	**31% decrease**	2% increase	nd
